# Supplementary material for: Assessing the person-centered care framework and assessment tool (PCC-AT) in HIV treatment settings in Ghana: A pilot study protocol
Source: PLoS One. 2024 Jan 5;19(1):e0295818. doi: 10.1371/journal.pone.0295818 (PMC10769038; doi:10.1371/journal.pone.0295818)
Supplement: S1 File — (DOCX) [file pone.0295818.s001.docx]

**Supplement 1: Selected and Alternate Study Health Facilities**

| **Name of facility** | **District** | **Administration/ Ownership** |
| --- | --- | --- |
| **Effia Nkwanta Regional Hospital** | Sekondi-Takoradi Metropolitan Assembly | Government |
| **Kwesimintsim Government Hospital** | Effia Kwesimintsim Municipal Assembly | Government |
| **Essikado Government Hospital** | Sekondi-Takoradi Metropolitan Assembly | Government |
| **Shama Health Center** | Shama | Government |
| **2^nd^ Medical Reception Station (2MRS)** | Effia Kwesimintsim Municipal Assembly | Quasi-Government |
| **Facilities for Pilot and replacement** | | |
