# Supplementary material for: Assessing the person-centered care framework and assessment tool (PCC-AT) in HIV treatment settings in Ghana: A pilot study protocol
Source: PLoS One. 2024 Jan 5;19(1):e0295818. doi: 10.1371/journal.pone.0295818 (PMC10769038; doi:10.1371/journal.pone.0295818)
Supplement: S2 File — (DOCX) [file pone.0295818.s002.docx]

**Supplement 2: FGD Guide**

**Interview Guide: Focus Group Discussions with HCW**

***Introduction:***

Hello, my name is ___ and I am a researcher for JSI conducting a study on person-centered care tool validation.  Now that we’ve conducted the tool and taken a look at the results, we would like to discuss the process and results with you:

A few reminders, these can be repeated if needed:

**Purposes of tool:**

The purpose of this tool  that you participated in earlier is to measure person-centered care (PCC) in a facility setting. This tool is designed to enable organizational learning, foster team sharing, and encourage reflective self-assessment within organizations. Recognizing that delivering PCC is a process and can take time, the use of the tool results in concrete action plans to provide organizations with a clear organizational development road map.

**What we did:**

As a summary of the process we just went through; we went through our PCC tool and selected options under each subdomain or topic in discussion that were most applicable to your facility. After selecting all the options, you received a score for each subdomain, with 1 being a low score indicating areas for improvement while 5 indicates a higher score in areas your facility excels in.

***Questions for HCW team:***

**Information**

Can you state your job title?

**Reaction to Process**

1. How long did it take to run through this?  Was this enough time? Too long?
2. Did you struggle to understand certain instructions or words in the tool without explanation?  How would you improve the instructions, or phrase the performance expectations, to be more clear or understandable?
3. Did you find the tool useful or helpful? If yes, how?
4. What changes would you make to the tool to make it more useful or helpful for the facility in delivering PCC?
5. Is this tool and process filling a need not already met? If yes, how?
6. Do you think this tool will help in improving your overall performance?
   1. If yes, can you envision using this tool routinely?
   2. If yes, how often do you think it would be feasible to use this tool and process?

**Reaction to Content**

1. What topics or themes that are important to PCC would you add? Are there any topics that you would remove?
2. Is this way of thinking about PCC useful? Why or why not?
3. Will this tool help modify or improve the way you deliver PCC? If yes, how?

*****Note to facilitator: Share results with the team, by domain and subdomain, then guide in an action planning session.*****

**Reaction to Results**

1. Were you surprised by any scores you received? Why or why not? If yes, which of the scores were you most surprised by?
2. Were you surprised by any actions identified through the action planning process?  Why or why not?  If yes, which of the actions were you most surprised by?
3. Do you feel you can implement or achieve the actions that have been suggested as next steps?
4. Do you feel some of the identified actions are out of the facility’s control? If yes, which ones?
5. How useful did you find the action planning process?

**Impressions of PCC at facility**

*Person centered care is described as…*Person-centered care (PCC) is a component of ‘quality of care’ that includes but moves beyond clinical quality of care to include concepts such as convenience, making services supportive and accessible, providing friendly services to diverse populations, and engaging communities and stakeholders.

1. Do you feel this facility delivers PCC services? Do you feel this facility has been able to meet clients expectations?
2. How would you rate your overall performance in regard to PCC? Do you feel there are areas for improvement?
