# Supplementary material for: Assessing the person-centered care framework and assessment tool (PCC-AT) in HIV treatment settings in Ghana: A pilot study protocol
Source: PLoS One. 2024 Jan 5;19(1):e0295818. doi: 10.1371/journal.pone.0295818 (PMC10769038; doi:10.1371/journal.pone.0295818)
Supplement: S3 File — (DOCX) [file pone.0295818.s003.docx]

**Supplement 3: KII Guide**

**Interview Guide: Key Informant Interviews with PLHIV**

***Introduction:***

Hello, my name is ___ and I am a researcher for JSI conducting a study on person-centered care tool validation.

We would like to take a moment of your time to get an overall sense of the care you receive here as well as your reactions to the results of an assessment we recently completed at this facility.

**Instructions:** review participant sheet and consent form

**Eligibility question:**

Are you a current client of this facility receiving HIV care/treatment?

**What we did:**

We sat with key staff at this facility and reviewed a set of questions that are intended to measure person centered care at this facility. Person-centered care (PCC) is a component of ‘quality of care’ that moves beyond clinical quality of care to include concepts such as convenience, making services supportive and accessible, providing friendly services to diverse populations, and engaging communities and stakeholders.

We would now like to ask you a few questions about your experience accessing care at this facility.

***Questions for PLHIV: (clients, KII)***

**Demographic information of client:**

- **Age**
- **Gender**
- **What year/when were you first diagnosed with HIV?**
- **What year/when did you begin HIV treatment?**
- **When did you first begin accessing HIV treatment services at this facility?**

**Impressions of PCC at facility:**

- *Person centered care is described as*… Person-centered care (PCC) is a component of ‘quality of care’ that moves beyond clinical quality of care to include concepts such as convenience, making services supportive and accessible, providing friendly services to diverse populations, and engaging communities and stakeholders… *Do you feel you receive PCC services at this facility?*
- What do you think of the care you receive at this facility?

1. What aspects do you like the most?
2. What areas do you feel need improvement?

**Experience of care received:**

Domain: Staffing

- *Subdomain: Composition*
  1. At this facility, have you been offered all recommended case services – clinical, counseling, laboratory, pharmacy, and case management? (*probe: do you usually have access to all your required clinical and psychosocial needs?)*
  2. Have you ever been offered supportive peer support? Do you have access to   any other form of counseling or support in your community? *(probe: expert client, navigators from the community)*
  3. On a scale of 1-4 (1= strongly disagree and 4= strongly agree), how important is staff composition to you? Are there any actions the facility could take within this subdomain to better meet your needs?
- *Subdomain: Availability*
  1. Are clinical, counseling, laboratory, pharmacy, and case management staff present when you visit the facility? (*probe: are the staff available when you need them?*)
  2. Do service providers at this facility ask you questions about your health and lifestyle? (*probe: would you say HCWs in this facility are sensitive to your general well-being?*)
  3. Do service providers at this facility give you time to ask questions? (*probe: would say HCWs in this facility are open to your views and anxieties?*)
  4. On a scale of 1-4 (1= strongly disagree and 4= strongly agree), how important is staff availability to you? Are there any actions the facility could take within this subdomain to better meet your needs?
- *Subdomain: Competency*
  1. Do service providers at this facility speak to you in a way that you understand? Do you experience any communication barriers? (*note: cultural and linguistic proficiency*)
  2. Do service providers at this facility inform you of your rights as a client here (for example, that you have equal access to services)?
  3. Do you feel providers at this facility offer unbiased, tolerant or judgment free services? Do service providers in this facility make you feel accepted?
  4. On a scale of 1-4 (1= strongly disagree and 4= strongly agree), how important is staff competency to you? Are there any actions the facility could take within this subdomain to better meet your needs?
- *Score consistency and reliability check:*
  1. After discussing all of these aspects of staffing, how would you rate the health facility’s performance in this area on a scale of 1-4? 1 = very poor, 2 = poor, 3= good, 4= very good.

Domain: Service Provision

- *Subdomain: Client Feedback Mechanisms*
  1. Have you ever been asked to provide feedback at this facility? If yes, what kind? (*probe: suggestion box, call-in number, post-care survey, or participation in a committee or meeting to give feedback on services to facility staff*)
  2. [If yes to the above], have you noticed any actions taken based on your feedback?
  3. On a scale of 1-4 (1= strongly disagree and 4= strongly agree), how important are client feedback mechanisms to you? Are there any actions the facility could take within this subdomain to better meet your needs?
- *Subdomain: Service Efficiency and Integration*
  1. In addition to your HIV care, have you ever been offered or referred for other services? (*probe: TB, FP, MNCH, NCD, mental health and substance use services*)
  2. Do you feel that you have privacy when speaking with provider(s) at this facility, or do you worry that you can be overheard or seen by others?
  3. When you arrive for services at this facility, are you typically seen within 30 minutes, or do you have to wait a long time?
  4. On a scale of 1-4 (1= strongly disagree and 4= strongly agree), how important is service efficiency and integration to you? Are there any actions the facility could take within this subdomain to better meet your needs?
- *Subdomain: Convenience and Access*
  1. Does the facility offer flexible timing for services? (*probe: during weekends or evenings*)
  2. Can you obtain medication refills at other locations? (*probe: mobile/outreach/community ART, private pharmacy, home delivery, other special locations*)
  3. On a scale of 1-4 (1= strongly disagree and 4= strongly agree), how important is convenience and access to you? Are there any actions the facility could take within this subdomain to better meet your needs?
- *Score consistency and reliability check:*
  1. After discussing all of these aspects of service provision, how would you rate the health facility’s performance in this area on a scale of 1-4? *1 = very poor, 2 = poor, 3= good, 4= very good.*

Domain: Direct Client Support

- *Subdomain: Psychosocial Support*
  1. Have you ever been offered peer support (by someone in your community and/or another person living with HIV)?
  2. Have you ever been asked about or treated for intimate partner violence?
  3. On a scale of 1-4 (1= strongly disagree and 4= strongly agree), how important is psychosocial support to you? Are there any actions the facility could take within this subdomain to better meet your needs?
- *Subdomain: Logistical Support*
  1. Has the facility ever asked you about your potential barriers to HIV care? (*probe: lack of transport, childcare barriers, food insecurity, among others*)
  2. Have you ever been offered transport, childcare, or food-related support from the facility?
  3. On a scale of 1-4 (1= strongly disagree and 4= strongly agree), how important is logistical support to you? Are there any actions the facility could take within this subdomain to better meet your needs?
- *Subdomain: Client Agency*
  1. Has the facility ever offered you information about HIV through a brochure, pamphlet, mobile phone, radio, TV or other formats?
     - If yes, were they easy to understand? (*probe: language, images/graphics, simple terminology*)
  2. If you wanted to report problems with accessing services, could you report at the facility? Do they have a reporting procedure or system?
  3. On a scale of 1-4 (1= strongly disagree and 4= strongly agree), how important is client agency to you? Are there any actions the facility could take within this subdomain to better meet your needs?
- *Subdomain: Digital Client Support Tools*
  1. Does the facility provide appointment reminders and scheduling services by phone or online?
  2. Does the facility provide reminder phone calls about adhering to treatment? For example, will they call you if your medication is not collected?
  3. Can you ask medical questions or access your test results and other health information from the facility over the phone or online?
  4. On a scale of 1-4 (1= strongly disagree and 4= strongly agree), how important are digital client support tools to you? Are there any actions the facility could take within this subdomain to better meet your needs?
- *Score consistency and reliability check:*
  1. After discussing all of these aspects of direct client support, how would you rate the health facility’s performance in this area on a scale of 1-4? *1 = very poor, 2 = poor, 3= good, 4= very good.*

**Closing:**

Are there any other activities or ways to make services friendlier, more convenient, supportive, accessible, friendly or acceptable, accessible or convenient that we have not yet discussed? If yes, what are they?
