# Supplementary material for: Assessing the person-centered care framework and assessment tool (PCC-AT) in HIV treatment settings in Ghana: A pilot study protocol
Source: PLoS One. 2024 Jan 5;19(1):e0295818. doi: 10.1371/journal.pone.0295818 (PMC10769038; doi:10.1371/journal.pone.0295818)
Supplement: S4 File — (DOCX) [file pone.0295818.s004.docx]

**Supplement 4: Informed Consent for Health Facility Staff**

**What is PCC?**

Person-centered care (PCC) is a component of ‘quality of care’ that moves beyond clinical quality of care to include concepts such as support, respect, and autonomy. Evidence demonstrates that PCC approaches lead to improvements across the HIV care continuum.

**What is this tool?**

JSI developed a tool to measure PCC in a facility setting. The approach used to develop the tool is founded on the understanding that discrete performance expectations reduce ambiguity, simplify assessment, and track progress on specific performance enhancements.

**Who are we?**

As an implementing partner of HIV service delivery programs, JSI advances person-centered care for HIV treatment. We are interested in working with you to refine the tool to improve facilities ability to deliver PCC.

**What are we doing today?**

Facilitators will spend approximately one day in each facility conducting the tool application. Focus group discussions (FGD) will be used to gather feedback from healthcare workers (HCW) at each facility. The FGDs will be run with the same set of staff that completed the tool and last approximately 45-60 minutes. The research lead will guide participants through a series of questions that are intended to obtain information on the process, results and other insights. Participants will speak with researchers on their takeaways from using the tool, any surprising findings that were found while running through the tool, and other comments such as disagreements or feedback on tool usage and wording.

**What happens in this study?**

As part of the study, you will be participating in two parts, 1) participation in the guided discussion to complete the tool and 2) 45 minute to one (1) hour long focus group discussion to examine the results of the tool, develop a set of actions items based on the results and reflect on the process. The questions will be focused on gaining a deeper understanding of your experience and perceptions of providing PCC services to your clientele.

The discussion will be guided by an external facilitator. The team will note your responses. We will be recording the interview so that we don’t miss any of the important points raised during

our conversation. However, if you choose not to be recorded, we will only take notes instead.

If you agree to participate, we will request that you be part of a group discussion with your staff members who completed the assessment.

**Can I refuse to be part of the study?**

It is important for you to know that participation in this study is voluntary, and you have the right to skip any questions you don’t feel comfortable answering or discontinue the interview at any point. Your name or any personal information will not be noted or be linked with any data in any way.

**What are the benefits of being in this study?**

Following the conclusion of the study, it is expected that evidence collected will allow our research team to improve upon the measure of PCC. Results and feedback will also help the team improve the tool and framework to be used as a guide for HIV treatment centers. The tool will also help facilities improve PCC processes and improve patient care and delivery. We hope that the participation of each clinic in the assessment will help them also improve upon delivering PCC.

**What are the risks of being in this study?**

There are no known risks or dangers to you for being involved in this study; if some of the questions make you uncomfortable feel free to decline to answer any questions you don’t wish to respond to. You may withdraw from the study at any time or refuse to participate in any part of the study.

**Confidentiality**

All information obtained from you will be kept confidential. No one outside the research team will be given access to any of your information. We will not use your name in any report of this project. Additionally, your name will not be used/recorded in any recording, note or papers; instead we will use codes. Once information that identifies you has been removed, the remaining information you provide may be shared publicly or with third parties, without additional informed consent from you or your legal representative.

*For Participation in FGDs*

You can help us to maintain confidentiality by refraining from sharing with others who are not in this study or discussing with them anything you have said in the group. We are also requesting you to refrain from sharing any information about who is the group or what they have talked about. We are however unable to guarantee that all those in the group will avoid disclosing information to others.

CONSENT FORM

STUDY TITLE: JSI: Person Centered Care Assessment Tool Validation

PARTICIPANTS’ STATEMENT

I acknowledge that I have read or have had the purpose and contents of the Participants’ Information Sheet read and all questions satisfactorily explained to me in a language I understand (……*name of language*). I fully understand the contents and any potential implications as well as my right to change my mind (i.e. withdraw from the research) even after I have signed this form.

I voluntarily agree to be part of this research.

Name of Participant…………………………..

Participants’ Signature ……………………...OR Thumb Print……………………………

Date:………………………………….
