# Supplementary material for: Assessing the person-centered care framework and assessment tool (PCC-AT) in HIV treatment settings in Ghana: A pilot study protocol
Source: PLoS One. 2024 Jan 5;19(1):e0295818. doi: 10.1371/journal.pone.0295818 (PMC10769038; doi:10.1371/journal.pone.0295818)
Supplement: S5 File — (DOCX) [file pone.0295818.s005.docx]

**Supplement 5: Informed Consent KII**

**What is PCC?**

Person-centered care (PCC) is a component of ‘quality of care’ that includes, but moves beyond clinical quality of care to include concepts such as support, respect, and autonomy. Evidence demonstrates that PCC approaches lead to improvements across the HIV care continuum.

**Who are we?**

As an implementing partner of HIV service delivery programs, JSI advances person-centered care for HIV treatment. We are interested in working with you to refine our tool to improve facilities’ ability to deliver PCC. JSI’s project here in Ghana is called The Care Continuum and works with the Government of Ghana to implement innovative strategies including index testing, contact tracing and testing, targeted outreach testing services, and use of case managers to identify, link and support clients to antiretroviral therapy (ART). The project is also championing ART adherence, generating demand to know one’s viral load, and creating reminder systems for people living with HIV.

**What are we doing? (Purpose)**

Facilitators will spend time with people living with HIV (PLHIV) to conduct key informant interviews (KII) to find out if the person-centered care framework and the results of the tool resonate with clients who have received services from this facility. PLHIV can expect to be asked questions about previous experiences in this facility and attaining care as a PLHIV. Feedback and responses from these questions are aimed to help guide facilities to improve on areas that received low scores and emphasize areas that received high scores.

**What happens in this study?**

As part of the study, you will be participating in a key informant interview lasting approximately 30-45 minutes. In each interview, we will ask you a few questions about your experience and satisfaction with your clinic. The interview questions will be focused on gaining a deeper understanding of your experience of care from this facility.

The interview will be conducted by a researcher. The team will note your responses. We

will be recording the interview so that we don’t miss any of the important points raised during

our conversation. However, if you choose not to be recorded, we will only take notes instead.

**Can I refuse to be part of the study?**

It is important for you to know that participation in this study is voluntary, and you have the right

to skip any questions you do not feel comfortable answering or discontinue the interview at any

point. Your name or any personal information will not be noted or be linked with any data in any

way. If you do not want to participate, please know that there will be no impact on receiving any

future health services.

**What are the benefits of being in this study?**

Following the conclusion of the study, it is expected that evidence collected will allow our research team to improve upon the measure of PCC. Results and feedback will also help the team improve the tool and framework to be used as a guide for HIV treatment centers. The tool will also help facilities improve PCC processes and improve patient care and delivery. We hope that the participation of each clinic in the assessment will help them also improve upon delivering PCC.

**What are the risks of being in this study?**

There are no known risks or dangers to you for being involved in this study; if some of the

questions make you uncomfortable feel free to decline to answer any questions you do not wish

to respond. You may withdraw from the study at any time or refuse to participate in any part of

the study.

**Confidentiality**

All information obtained from you will be kept confidential. No one outside the research team

will be given access to any of your information. We will not use your name in any report of this

project. Additionally, your name will not be used/recorded in any recording, note or papers;

instead we will use codes. Once information that identifies you has been removed, the remaining information you provide may be shared publicly or with third parties, without additional informed consent from you or your legal representative.

CONSENT FORM

STUDY TITLE: JSI: Person Centered Care Assessment Tool Validation

PARTICIPANTS’ STATEMENT

I acknowledge that I have read or have had the purpose and contents of the Participants’ Information Sheet read and all questions satisfactorily explained to me in a language I understand (……*name of language*). I fully understand the contents and any potential implications as well as my right to change my mind (i.e. withdraw from the research) even after I have signed this form.

I voluntarily agree to be part of this research.

Name of Participant…………………………..

Participants’ Signature ……………………...OR Thumb Print……………………………

Date:………………………………….
