# Supplementary material for: Assessing the person-centered care framework and assessment tool (PCC-AT) in HIV treatment settings in Ghana: A pilot study protocol
Source: PLoS One. 2024 Jan 5;19(1):e0295818. doi: 10.1371/journal.pone.0295818 (PMC10769038; doi:10.1371/journal.pone.0295818)
Supplement: Attachment — Submitted filename: Plos Jul2023.docx [file pone.0295818.s006.docx]

*The person-centered care assessment tool (PCC-AT): A study protocol to assess*

*content validity, score consistency and reliability, and feasibility in HIV treatment*

*settings in Ghana*

July 14, 2023

Patient-centered care is critical to improving the well-being of people living with HIV. This manuscript tackles a vital topic in HIV service delivery. Its strengths include:

- Attempt to create a scale to measure patient-centered care.
- Use of a systematic review-informed theoretical framework.
- Sampling across multiple facilities in Ghana.

There are a few areas that need strengthening.

Introduction

o The authors describe that they collected feedback on the domains of the scale at the IAS and ICASA conferences. It would be helpful to include the method of data collection. Figure 2 shows that they used FGDs and surveys from IAS and ICASA. However, these methods are not mentioned in the introduction.

o The study objectives need to be rewritten as clear sentences instead of short phrases. For example: Determine the feasibility of PCC-AT instead of “PCC-AT feasibility.”

o Relating to the first objective, is it a re-evaluation of the content validity of the PCC-AT, given the data they collected from the IAS and ICASA? It is hard to tell because details of what was collected in the FGDs and surveys at the IAS meeting ICASA are scarce.

o One argument advanced in this paper is that PCC may differ across contexts or clinical settings. So, what context is this scale designed for? Ghana? All African countries? Global tool? Why was content validity data from the first round collected from many countries, and now data is being collected from Ghana alone?

Materials and Methods

o The sampling of health workers and participants could be more robust and not tied to the day of data collection. It appears like, if the research team shows up, whoever is there is who they interview, particularly health workers. Because the scale includes multiple domains, they will need to be more purposeful in selecting participants. If specific groups of participants are away on the data collection day, should more attempts be made to recruit them?

o One of the domains is leadership, but in the list of participants to be interviewed, there appears to be no purposeful attempt to interview leaders, heads of departments, units, or staff.

o The justification for seven people needs to be clarified. Any reason why? Particularly as they also desire a balance in gender and years of experience.

o Is there a reason why adolescents are excluded?

o It also needs to be clarified why those working less than 12 months are excluded. Wouldn’t sampling them give a continuum of years of experience?

o It would be helpful for the authors to include cognitive interviewing to understand the wording of the items within the scale. Ghana has many languages, and research assistants sometimes cannot read and write in the local language. They orally translate English into the local language. So they must figure out the precise wording of items and assess how participants understand the questions.

o Having worked on scales in Ghana, there is a real challenge when a Likert scale is used as a response format. People sometimes have difficulty distinguishing between a range of categories. In addition, there are often responses where participants are non-committal to any specific response. They will say: “It is “OK”—meaning neither good nor bad. Thus, there needs to be a neutral response to the options provided to participants. It would be compelling and helpful if they also asked about the scoring format for the scale.

o Line 200: “Once the scoring is complete, each PCC-AT pilot will culminate in an action planning process, led by the facilitator, based upon PCC-AT scores.” Could they clarify who the participants here are? What will they be planning?

o Line 179—relating to the analysis “ feasibility of the PCC-AT through a pilot among health facility staff followed by FGDs.” I was unclear if the pilot is different from the FGDs. Are the health workers scoring first, and then the data is used for the FGDs, or the FGDs is part of the pilot testing, or are they done in the same session?

o The challenge is often finding a place at the health facility to interview privately. Is there a designated room for this?

o Could they be specific on the statistical analysis to assess the “concordance reliability by comparing the PCC-AT scores assigned by health facility staff to the perspectives of PLHIV key informants”? What statistics would be reported? How would concordance be evaluated?

o Perceived and enacted stigma is integral to the lived experience of people living with HIV. Is there a specific sub-domain that captures stigma?

o Would there be room in the methodology for iteration? Like participants would suggest various modifications. Would those modifications be made, and feedback solicited on them before the scale is finalized? It seems like all the data would be collected and analyzed later. If there is room to start with a few participants, refine, and sample again—like how Grounded Theory works—so that the final product is complete.
