# Supplementary material for: Assessing the person-centered care framework and assessment tool (PCC-AT) in HIV treatment settings in Ghana: A pilot study protocol
Source: PLoS One. 2024 Jan 5;19(1):e0295818. doi: 10.1371/journal.pone.0295818 (PMC10769038; doi:10.1371/journal.pone.0295818)
Supplement: Attachment — Submitted filename: Review_PLOS_Aug2023.docx [file pone.0295818.s007.docx]

**Summary:**

This article presents the protocol for a study assessing content validity, score consistency and reliability, and feasibility in HIV treatment settings in Ghana of a person-centered care assessment tool (PCC-AT). It is an original and timely study protocol with growing attention to person-centered care approaches. However, there are concerns, particularly to the methods and materials section, and the goal of the protocol, which reads as a mix of framework/theory development, measure assessment, and implementation assessment.

**Title:** The title is a bit misleading. I presumed based on the title that this protocol would focus on some standard quantitative psychometric approaches to assessing reliability. In addition, an entire objective of the study is focused on content validity of the PCC framework, not the PCC-AT.

**Introduction:**

- Page 4, line 87 says six countries but lists seven
- Page 5, line 110 says give countries but lists four

**Methods:**

- I am a bit unclear about the assessment of the content validity of the PCC framework and timing. I would think that assessment of the framework for content validity would proceed finalization of the PCC-AT (and any assessments of reliability or validity). Either a clarification of timing or further explanation is needed.
- Overall, some reorganization of this section would help to make it clear. For example, you could organize the different activities under the “Participants” sub-section by type of data collection, or by objective. Inclusion/exclusion criteria for each activity would also be easier to read if bulleted.
- The methods for the focus groups are not well described. Is it one per facility? How will they be convened? Any comment/justification on sample size?
- More explanation/justification of choice of methods to address the objectives is needed.
- Is the PCC-AT being administered to KII participants in full. This is unclear as described.
- Are the authors able to share the PCC-AT? Methods overall are hard to follow without that as a grounding instrument to look at. For example the authors say as part of the KII that participants will score each domain: is that part of the PCC-AT? What is the criteria for scoring a domain as “Very poor” “Poor”…etc? I can see how the authors are addressing content validity potentially, but more explanation is needed to say there is an assessment of reliability. I do not see a description of data collection or anlaysis that assess reliability (e.g. inter-rater, test-retest…etc.)
- Page 8, line 171: authors say they “will note commonalities between client responses to estimate how large a sample of clients are needed per facility to reach saturation”. Authors should consider the following suggestions to strengthen sample size justification:
  - Define/include appropriate citations for the principle of saturation.
  - Better explain how they will assess saturation to ensure it has been achieved.
- Page 8, line 175: please add additional details about the trained researchers (are they Ghanian, American, members of the investigative team?)
- Please add additional details on informed consent
  - What languages will the consent form be available in or will it be translated from English by the person obtaining consent?
  - What about people who cannot write?
  - What if people do not speak Twi or English (as noted under Materials) – will this be an exclusion reason?
- Please add more details (and relevant citations) to the qualitative analysis plan, at a minimum:
  - More description of codebook development
  - Software used or approach to applying codes
  - Domains you intend to structure analysis under
  - Will you do formal intercoder comparison? If so, describe methods and include citations. You not have to do intercoder comparisons, but if not, describe process for aligning coding and harmonizing interpretation of codes
  - More description of coding and code comparison/how discrepancies will be handled

**Discussion**

- Is there discussion of other practical study considerations not covered in other sections the authors could include here?
- Limitations:
  - This study is largely qualitative, but the limitations read as if written for a quantitative study (e.g. external validity). Authors should rather consider appropriate qualitative measures of rigor to comment on or add to this section.
- Dissemination
  - Please describe any dissemination plans to PLHIV and/or study participants
