# Supplementary material for: Assessing the person-centered care framework and assessment tool (PCC-AT) in HIV treatment settings in Ghana: A pilot study protocol
Source: PLoS One. 2024 Jan 5;19(1):e0295818. doi: 10.1371/journal.pone.0295818 (PMC10769038; doi:10.1371/journal.pone.0295818)
Supplement: Attachment — Submitted filename: 9.30.23_Response to Reviewers_Ghana Study Protocol.docx [file pone.0295818.s008.docx]

September 30, 2023

PONE-D-23-15863

The person-centered care assessment tool (PCC-AT): A study protocol to assess content validity, score consistency and reliability, and feasibility in HIV treatment settings in Ghana

Dear Dr. Hurley,

Thank you for your email, and for the opportunity to respond and revise the manuscript. We have incorporated each of the reviewers’ comments into a revised manuscript. For clarity, we have numbered each of the reviewers’ comments, and provided our responses in underlined text, with the relevant sections from the adapted paper in italics. We would like to thank the reviewers for their suggestions, which we hope have made for a more precise and stronger paper.

The Authors.

***Editor feedback:***

After reviewing the manuscript and the reviewer's comments, I do believe that addressing or clarifying each of the issues raised by reviewers would strengthen the paper. Both reviewers also suggested some strengthening and clarification of the qualitative methods and a reflection of qualitative limitations. I additionally recommend adding citations to the qualitative methods and reviewing the CORE-Q guidelines to add any details on qualitative data collection, analysis and/or rigor that are currently missing from the paper:  <https://www.equator-network.org/reporting-guidelines/coreq/>

Thank you for these suggestions. We have added further details to the Materials and Methods section based upon the CORE-Q guidelines. We have also added citations in regard to our sample size, how we anticipate achieving saturation, and/or mitigating for it as well as reporting results in line with the CORE-Q guidelines.

**Reviewer #1:**

Introduction

1. The authors describe that they collected feedback on the domains of the scale at the IAS and

ICASA conferences. It would be helpful to include the method of data collection. Figure 2 shows

that they used FGDs and surveys from IAS and ICASA. However, these methods are not

mentioned in the introduction.

Thank you very much for this comment. We have added that a focus group discussion was conducted to gather information from the 25 stakeholders. The same paragraph also contains information regarding how survey data was collected via tablets for people who visited the author’s booth and/or attended author presentations.

2. The study objectives need to be rewritten as clear sentences instead of short phrases. For

example: Determine the feasibility of PCC-AT instead of “PCC-AT feasibility.”

Thank you for the suggestion. We have re-worded each objective accordingly.

3. Relating to the first objective, is it a re-evaluation of the content validity of the PCC-AT, given

the data they collected from the IAS and ICASA? It is hard to tell because details of what was

collected in the FGDs and surveys at the IAS meeting ICASA are scarce.

Thank you for highlighting this. We have reiterated within the objective that the previous content validity assessments were limited to public health practitioners and not necessarily people with lived experience. Please see the additional text within objective 1: *“Gathering the perspectives of PLHIV is a critical measure given previous content validity assessments of the PCC framework were limited to public health practitioners.”*

4. One argument advanced in this paper is that PCC may differ across contexts or clinical

settings. So, what context is this scale designed for? Ghana? All African countries? Global tool?

Why was content validity data from the first round collected from many countries, and now data

is being collected from Ghana alone?

The PCC framework is based upon findings from the systematic review of the literature which was limited to countries across sub–Saharan Africa. Content validity was gathered from practitioners who work in various sub-Saharan African settings including Ghana, Uganda and Zambia. Those individuals providing feedback from the United States and Canada provided their insights based upon their experience working in multiple sub-Saharan African settings. The study team chose to implement the study in Ghana based upon the elements described in the manuscript. However, the next phase of our research will examine PCC-AT implementation in Zambia, which will help us determine if further contextual adaptations are required based upon country context.

Materials and Methods

5. The sampling of health workers and participants could be more robust and not tied to the day

of data collection. It appears like, if the research team shows up, whoever is there is who they

interview, particularly health workers. Because the scale includes multiple domains, they will

need to be more purposeful in selecting participants. If specific groups of participants are away

on the data collection day, should more attempts be made to recruit them?

Thank you for this comment. The study team in Ghana who supports these facilities with technical assistance, is familiar with the staff composition in the study clinics and was knowledgeable about the type of staff that would be available on any given day. In addition, the study team called in advance to ensure preparations were made for the right mix of staff to be available on the day of data collection to ensure that knowledgeable staff persons would be available relative to each PCC-AT domain.

6. One of the domains is leadership, but in the list of participants to be interviewed, there appears

to be no purposeful attempt to interview leaders, heads of departments, units, or staff.

The invitation letter to facilities specifies the type of staff who should be involved, and this includes department leadership. We have included this detail within the manuscript.

7. The justification for seven people needs to be clarified. Any reason why? Particularly as they

also desire a balance in gender and years of experience.

Thank you for this question. We have added the following explanation to the manuscript.

*Health facility staff: We will aim for a minimum of seven health facility staff members to participate in the PCC-AT pilot and FGDs at each health facility for a total of five pilots and FGDs. The number of FGDs aligns with recent research estimating sample sizes in focus group research to achieve saturation.(18) The number of staff participants selected to participate in each PCC-AT pilot and FGD is based upon the study team’s knowledge of staffing at study facilities and is consistent with the number of staff who will be available to participate on the study date while ensuring a mix of staff who can provide diverse perspectives based upon their roles.*

8. Is there a reason why adolescents are excluded?

We chose to include people aged 18 and older because they are able to independently provide consent without a parent/guardian. Often adolescents attend ART visits without their parent/guardian. If they were to be invited to participate, this would require them to travel back to their home to obtain consent before returning to the health facility. The study team felt that this was too onerous for potential key informants. We have added the following to the limitations section:

*We have also chosen to limit the study to people aged 18 and above due to parental consent challenges which limits the perspectives that will be gathered.*

9. It also needs to be clarified why those working less than 12 months are excluded. Wouldn’t

sampling them give a continuum of years of experience?

Thank you for this comment. We ultimately decided not to exclude staff working less than 12 months. This exclusion criteria has been deleted from the manuscript. The reason we initially chose to include a minimum work period was to make sure people had been at the facility long enough to be able to speak confidently about PCC services provided, however, ultimately, we chose to include all staff regardless of how long they have been employed at the facility.

10. It would be helpful for the authors to include cognitive interviewing to understand the wording

of the items within the scale. Ghana has many languages, and research assistants sometimes

cannot read and write in the local language. They orally translate English into the local language.

So they must figure out the precise wording of items and assess how participants understand the

questions.

Thank you for this constructive feedback. The key informant interviews have already been conducted (April, 2023). Study participants were given a choice of the language they would like to be interviewed in and study team members fluently speak the Akan languages including Twi and Fante which helped to avoid oral translation of English into the local languages. The questionnaires were pre-tested and reviewed with the Ghana team that provides technical assistance to these facilities for appropriateness.

11. Having worked on scales in Ghana, there is a real challenge when a Likert scale is used as a

response format. People sometimes have difficulty distinguishing between a range of categories.

In addition, there are often responses where participants are non-committal to any specific

response. They will say: “It is “OK”—meaning neither good nor bad. Thus, there needs to be a

neutral response to the options provided to participants. It would be compelling and helpful if

they also asked about the scoring format for the scale.

Thank you for these very insightful comments. We acknowledge the challenge with Likert scales in the study context. In addition to asking for a numerical response, our qualitative guide aims to supplement data from the Likert scales with further information that serves to provide a richer understanding of each key informant’s perspectives and experiences. We have added the challenge with Likert scales within this context as a potential study limitation.

12. Line 200: “Once the scoring is complete, each PCC-AT pilot will culminate in an action

planning process, led by the facilitator, based upon PCC-AT scores.” Could they clarify who the

participants here are? What will they be planning?

Thank you for this question. We have added the following to provide clarification.

*“Through the action planning process, as the final step of the PCC-AT process conducted among the same health facility staff, participants identify areas of low performance and strengthening activities that address performance gaps.”*

13. Line 179—relating to the analysis “feasibility of the PCC-AT through a pilot among health

facility staff followed by FGDs.” I was unclear if the pilot is different from the FGDs. Are the

health workers scoring first, and then the data is used for the FGDs, or the FGDs is part of the

pilot testing, or are they done in the same session?

Thank you for this point of clarification. The PCC-AT was piloted and then focus group discussions were conducted among the staff who participated in the PCC-AT pilot, to gather their insights on PCC-AT feasibility. We have changed the language to the following:

*“…and 3) feasibility of the PCC-AT through piloting the PCC-AT among health facility staff, followed by FGDs to assess staff perspectives on its implementation.”*

14. The challenge is often finding a place at the health facility to interview privately. Is there a

designated room for this?

The study team conducted the research at lunchtime and in the afternoon when there were less patients available to ensure that sufficient space was available and while not inconveniencing any patients. However, given the limited space in study clinics, the study team was required to use any available space including empty waiting rooms or treatment rooms.

15. Could they be specific on the statistical analysis to assess the “concordance reliability by

comparing the PCC-AT scores assigned by health facility staff to the perspectives of PLHIV key

informants”? What statistics would be reported? How would concordance be evaluated?

Thank you for this suggestion. We have added the following information to the manuscript.

*The study team will assess score consistency and reliability through a descriptive comparison of performance scores identified by health facility staff through the PCC-AT process against client-assigned performance scores for each PCC-AT domain, including average client scores by facility. This was important to better assess the concordance reliability between the providers scores and the actual lived experiences of PLHIV. A Spearman’s rank correlation will be calculated to assess the statistical relationship between health facility staff and client scores.*

16. Perceived and enacted stigma is integral to the lived experience of people living with HIV. Is

there a specific sub-domain that captures stigma?

Thank you for this comment. We agree with the reviewer that stigma is a critical aspect of PCC and is central to the experiences of people living with HIV. Under the competency sub-domain there are two performance expectations specific to stigma including:

- Facility staff have been trained within the last year in client rights, providing stigma and judgment free service provision.
- The facility holds staff accountable for respecting client rights and providing stigma and judgment free service.

Also, the client agency subdomain includes the following performance expectation:

- The facility has a documented process/procedure for clients to report any problem related to accessing services confidentially, including discrimination against them personally, including: 1) process/procedure is documented; 2) process/procedure is implemented; 3) there is evidence that feedback is reviewed, and action has been taken; and 4) there is evidence that feedback is reviewed and actions are taken in a systematic manner.

17. Would there be room in the methodology for iteration? Like participants would suggest various

modifications. Would those modifications be made, and feedback solicited on them before the

scale is finalized? It seems like all the data would be collected and analyzed later. If there is

room to start with a few participants, refine, and sample again—like how Grounded Theory

works—so that the final product is complete.

Thank you for this comment. Yes, FGDs focused on gathering specific staff insights around potential modifications that are required in addition to KIIs from clients. The study findings will be used to adapt the tool prior to its finalization. Because we now have the results, we know that no major modifications to the tool were required other than minor verbiage changes and definitions for some of specific PCC activity examples. Because the study team supports the health facilities where the PCC-AT will be implemented, the PCC-AT can undergo further modifications at any point following findings from future scale-up. Please see the following language:

*Information from the study will be used to further refine the PCC-AT including its content, indicators, scoring, and application in practice. Once finalized, the study team will collaborate with the 99 health facilities under the purview of JSI projects in Ghana to integrate the tool as a standard of care. The study team will also use findings from the Ghana study to inform a planned study in Zambia in August 2023 that will evaluate the relationship between a health facility’s PCC-AT score and key health indicators including antiretroviral treatment (ART) coverage, continuity in ART, and viral suppression.*

**Reviewer #2**

1. Title: The title is a bit misleading. I presumed based on the title that this protocol would focus

on some standard quantitative psychometric approaches to assessing reliability. In addition, an

entire objective of the study is focused on content validity of the PCC framework, not the PCC-

AT.

Thank you for noting this. To provide a more accurate description, we have changed the title to:

*Assessing the person-centered care framework and assessment tool (PCC-AT) in HIV treatment settings in Ghana: A pilot study protocol*

Introduction:

2. Page 4, line 87 says six countries but lists seven

Thank you for noting this. We have corrected it within the text.

3. Page 5, line 110 says give countries but lists four

Thank you for noting this. We have corrected it within the text.

Methods:

4. I am a bit unclear about the assessment of the content validity of the PCC framework

and timing. I would think that assessment of the framework for content validity would

proceed finalization of the PCC-AT (and any assessments of reliability or validity). Either

a clarification of the timing or further explanation is needed.

Thank you for noting this. The systematic review, which informed the initial PCC framework, was limited to our findings from the peer reviewed literature. The PCC framework provided the basis to develop the content for the PCC-AT. However, the study team was cognizant of the fact that the PCC framework had undergone content validation only by public health practitioners, but not by PLHIV. To ensure that we were including the perspectives of our most important audience (PLHIV) as part of this study, the team decided to integrate a content validation assessment into the study, to understand if the PCC framework, which provides the basis for the PCC-AT is relevant to the PCC needs of PLHIV. The PCC-AT which was piloted in this study is not the final tool. Any findings from the content validation exercise that would prompt changes to the PCC-AT will be incorporated prior to finalization of the PCC-AT.

To reflect this process more accurately, we have changed Figure 2, to read *“updated PCC-AT prepared for pilot study in Ghana”.*

We also added the following sentence to study objective 1: *Gathering the perspectives of PLHIV is a critical measure given previous content validity assessments of the PCC framework were limited to public health practitioners.*

5. Overall, some reorganization of this section would help to make it clear. For example, you could organize the different activities under the “Participants” sub-section by type of data collection, or by objective. Inclusion/exclusion criteria for each activity would also be easier to read if bulleted.

Thank you for this suggestion. We have added sub-headings for Health Facility Staff and Client Key Informants to help differentiate the audiences.

6. The methods for the focus groups are not well described. Is it one per facility? How will

they be convened? Any comment/justification on sample size?

Thank you for these questions. We have further explained in the manuscript that there will be one FGD per facility and added further detail about where FGDs will take place. We also explained that through discussions with the project team who is familiar with the study health facilities, aiming for a minimum of seven participants for the PCC-AT and FGDs is consistent with the number of staff who would be available to participate on the study date while ensuring a mix of staff who can provide diverse perspectives based upon their roles.

7. More explanation/justification of choice of methods to address the objectives is needed.

To provide further explanation/justification for the FGD and KII methods, we have added the following information:

*We chose to conduct FGDs among health facility staff given their proven value in healthcare research to facilitate group exploration of systems processes and services while promoting synergies among participants and opportunities to disagree (20). We chose to conduct KIIs among PLHIV key informants as a complementary measure to the FGDs. The individual structure of KIIs intends to protect clients’ confidentiality and increase their confidence to share information on their experiences and to generate suggestions and recommendations in a private setting (21).*

8. Is the PCC-AT being administered to KII participants in full. This is unclear as described.

Thank you for this question. We have clarified in the text that the KII participant interviews are conducted via a KII study guide (provided in a Supplement Guide 3). The KII guide required the study team to gather information from clients on their perceptions of domain scores based upon their subjective experiences whereas the PCC-AT was used with health facility staff to measure scores through the performance expectations.

9. Are the authors able to share the PCC-AT? Methods overall are hard to follow without that as a grounding instrument to look at. For example, the authors say as part of the KII that participants will score each domain: is that part of the PCC-AT? What is the criteria for scoring a domain as “Very poor” “Poor”…etc? I can see how the authors are addressing content validity potentially, but more explanation is needed to say there is an assessment of reliability. I do not see a description of data collection or analysis that assess reliability (e.g. inter-rater, test-retest…etc.).

Thank you for this suggestion. We have added the following information to the manuscript.

*The study team will assess score consistency and reliability through a descriptive comparison of performance scores identified by health facility staff through the PCC-AT process against client-assigned performance scores for each PCC-AT domain, including average client scores by facility. This was important to better assess the concordance reliability between the providers scores and the actual lived experiences of PLHIV. A Spearman’s rank correlation will be calculated to assess the statistical relationship between health facility staff and client scores.*

We are not able to share a draft PCC-AT at this time, we have attempted to provide strong clarity on the key domains (Figure 1) and the subdomain questions in the KII (Supplement 3) which provides a good amount of detail on the draft PCC-AT. Scoring those domains are based on the client’s experiences at the facility. We have added that this could be a bias in the limitations section.

10. Page 8, line 171: authors say they “will note commonalities between client responses to estimate how large a sample of clients are needed per facility to reach saturation”. Authors should consider the following suggestions to strengthen sample size justification:

o Define/include appropriate citations for the principle of saturation.

o Better explain how they will assess saturation to ensure it has been achieved.

Thank you for this important suggestion. We have included a citation for how we determined saturation and the following within the manuscript:

*Drawing upon previous research (17), we will determine that we have reached saturation when there is no new data after two consecutive interviews. At minimum, we will aim to include four clients per facility so as not to disrupt client flow.*

11. Page 8, line 175: please add additional details about the trained researchers (are they

Ghanaian, American, members of the investigative team?)

Thank you for noting this. We have specified the following:

*This study team includes two trained female Ghanaian researchers who are independent consultants who are trained/certified in ethical research and are familiar with PCC concepts as well as the PCC-AT.*

12. Please add additional details on informed consent

o What languages will the consent form be available in or will it be translated from

English by the person obtaining consent?

o What about people who cannot write?

o What if people do not speak Twi or English (as noted under Materials) – will this

be an exclusion reason?

We have updated the manuscript to provide details for each of these items.

Informed consents were available in English and translated by the study team into the client’s preferred language. Fingerprints were provided in place of signatures for people unable or unwilling to write. Study team members were instructed to be sure to let all people know they spoke all three languages and were comfortable in all.

13. Please add more details (and relevant citations) to the qualitative analysis plan, at a

minimum:

o More description of codebook development

o Software used or approach to applying codes

o Domains you intend to structure analysis under

o Will you do formal intercoder comparison? If so, describe methods and include

citations. You do not have to do intercoder comparisons, but if not, describe process for aligning coding and harmonizing interpretation of codes

o More description of coding and code comparison/how discrepancies will be

handled

Thank you for requesting further details regarding our qualitative analysis plan. We have updated the text to include the following:

*Qualitative analysis will derive from discussions associated with PCC-AT pilot and the resulting action plans, FGDs, and KIIs and consist of thematic analysis of findings. For each data source, one researcher will examine findings and develop an initial codebook using NVIVO software which will be shared with a second researcher. PCC-AT pilot and action planning discussions, and FGD and KII transcription data will be separately analyzed by the two qualitative researchers using the codebook as the foundation while adding new codes and sub-codes as themes emerge through analysis. Illustrative codes the study team anticipates for the FGDs will include length of time, structure, comprehensibility, and suggestions for changes. For the KIIs, illustrative codes that the study team anticipates will include staff accessibility, wait times, client feedback, ART dispensation, and logistical support. After both qualitative researchers separately analyze the data, they will meet to compare data included under each code and sub-code. Differences will be identified and discussed. If consensus is not reached, a third study team member will help to make final determinations.*

Discussion

14. Is there discussion of other practical study considerations not covered in other sections the authors could include here?

Thank you for this suggestion, we have further rounded out the discussion to include further limitations that the reviewers have noted and provided further details on the dissemination plan.

15. This study is largely qualitative, but the limitations read as if written for a quantitative study (e.g., external validity). Authors should consider appropriate qualitative measures of rigor to comment on or add to this section.

Thank you for this suggestion. We have added the following to the limitations section of the discussion.

*In addition, the researcher’s presence during qualitative data collection may affect/bias participant’s responses and clients who consent to participate in the study may offer different inputs than any clients who may decline to participate. As convenience sampling was used it can result in a biased and unrepresentative of the population in question. The perceptions of the clients were recorded on a four-point Likert Scale which may not sufficiently capture the perspectives and experiences of key informants. The quality of the data collected is dependent on the researcher’s skills, background, and personal biases. In effort to improve the rigor all interviews were done by trained Ghanian researchers fluent in the three main languages in Ghana. We have also chosen to limit the study to people aged 18 and above due to parental consent challenges which limits the perspectives that will be gathered. Though client key informants will be reassured that their responses will be confidential and that there will be no positive or negative consequences to their participation, there remains a risk of response bias if clients do not feel secure sharing information with the study team. Similarly, health facility staff may not openly share their perspectives in a focus group setting. Qualitative data captured via the key informant guide aims to overcome this challenge through further drawing out information via one-on-one interviews.*

Dissemination

16. Please describe any dissemination plans to PLHIV and/or study participants

Thank you for this suggestion. We have also added that findings will be shared with the HIV Technical Working Group, Ghana AIDS Commission Research, Monitory and Evaluation Committee and the HIV community during the National HIV and AIDS Conference (NHARCON).
